# Factors associated with musculoskeletal pain in the past 12 months amongst female miners in a South African goldmine

**DOI:** 10.4102/sajp.v77i1.1476

**Published:** 2021-01-18

**Authors:** Khathutshelo P. Kabongo, Saloshni Naidoo

**Affiliations:** 1 Department of Mineral Resources, Pretoria, South Africa; 2 Discipline of Occupational and Environmental Health, School of Nursing and Public Health, College of Health Sciences, University of KwaZulu-Natal, Durban, South Africa; 3 Discipline of Public Health Medicine, School of Nursing and Public Health, College of Health Sciences, University of KwaZulu-Natal, Durban, South Africa

**Keywords:** women, musculoskeletal pain, mining, psychosocial risks, physiotherapy

## Abstract

**Background:**

Mineworkers, including females, are prone to work-related musculoskeletal disorders and these disorders are not well reported amongst female mineworkers in South Africa.

**Objective:**

This study aimed to identify the factors associated with the presence of musculoskeletal pain over a 12-month period amongst female mineworkers (*n* = 225) in a South African goldmine.

**Method:**

Female mineworkers’ responses to the presence of musculoskeletal pain over the previous 12-month period and their exposure to physical and psychological factors were elicited using a standardised questionnaire. Descriptive and multivariable analytical statistics were conducted to test for associations between physical and psychological factors and the presence of musculoskeletal pain over the previous 12 months.

**Results:**

The median age of participants was 40 years, with 50.22% of participants reporting the presence of musculoskeletal pain over the previous 12 months. On multivariable analysis, education level and good to excellent health status were protective against the existence of pain during the previous 12 months. Participants’ perceived ratings of experiencing moderate-to-high pain intensities were significantly associated with reports of pain over the past 12 months.

**Conclusion:**

These findings may raise awareness amongst physiotherapists and occupational medicine physicians about the factors associated with musculoskeletal pain amongst female mineworkers, which they can then use in managing and developing interventions aimed at improving the physical health of female mineworkers.

**Clinical implications:**

Our study highlights the need for medical surveillance of musculoskeletal pain in female mineworkers. A cohort study in a larger study population and across several mines will build on the existing cross-sectional information and help inform workplace interventions for female mineworkers.

## Introduction

Mining, which is physically intense and psychologically demanding work, has been a male-dominated setting for several centuries. A high prevalence of musculoskeletal disorders (MSDs) has been reported amongst miners in several countries (Bandyopadhyay, Dev & Gangopadhyay 2012; Widanarko et al. 2015b; Xu et al. 2012a). Low back pain reported amongst miners in various countries has ranged from 23% in Indonesian miners to 78% in Turkish miners (Bandyopadhyay et al. 2012; Bio et al. 2007; Sarikaya et al. 2007; Widanarko et al. 2013; Xu et al. 2012a). Pain in other anatomical sites such as the neck, shoulder, wrist and leg has also been reported amongst Indian coal miners (Bandyopadhyay et al. 2012). The nature of underground mining activities presents several physical and psychosocial risks for workers, which have been associated with work-related MSDs (Widanarko et al. 2015a). Physical risk in mining activities associated with MSDs includes heavy lifting, working in awkward postures, vibration exposure and repetitive movements (Bio et al. 2007; Kunda 2008; Widanarko 2013; Xu et al. 2012a). Psychosocial factors associated with MSDs include high psychological demands and work stress, low-decision latitude, lack of social support and poor job satisfaction (Fjell et al. 2007; Widanarko et al. 2015b).

Increasingly, women, who have started to form a fundamental part of mining activities in several countries, are involved in both the artisanal and formal mining industries. In South Africa, a country with a long mining history, changes in the political climate and employment legislation have seen the entry of women into the formal mining sector since 1996. In 2018, women working in mining constituted approximately 12% of the workforce on South African mines (Minerals Council of South Africa 2018). Research of MSDs amongst miners in South Africa has focussed on the male-dominated worker populations with Dias (2014) studying miners working in coal, gold and platinum mines in 2014, reporting an annual MSD incidence of 2.7% (Dias 2014).

The majority of studies performed thus far amongst women working in mining in South Africa have focussed on gender perspectives of the workplace and the challenges encountered by women entering this sector (Benya 2009; Botha & Cronje 2015; De Klerk 2012; Mokotong 2016). Thus, our study aimed to identify the factors associated with the presence of musculoskeletal pain in the past 12 months amongst female miners in a South African gold mine. A better understanding of the factors associated with the presence of musculoskeletal pain in the past 12 months amongst female miners may help health practitioners, such as occupational medicine physicians and physiotherapists, in their management of women from the mining industry who present to them for care.

## Method

Our epidemiological observational cross-sectional study was conducted between August 2016 and January 2017 in a single gold mine in Gauteng, South Africa. This mine has the largest workforce of all gold mines in South Africa, which comprises 6482 miners, with 589 of them being women. The mine has three major shafts where women work. These women work underground as labourers as well as professionals such as engineers and geologists. The mine has its in-house medical services, including primary healthcare, occupational healthcare, first aid rooms and shaft clinics for workers. The mine clinic offers primary and secondary medical services, 24 h a day, 7 days a week to all employees in the mine. A complete list for the study population was obtained from the Human Resource Department of the mine. In total, 589 females were working on the mine, with 553 being black South Africans and 33 white South Africans and three females of mixed race at the time of our study.

Our study sample included all the females of black South African origin (*n* = 553). In total, 423 (76.49%) females of black South African origin work underground, whilst 130 (23.50%) work on the surface ((surface normal (*n* = 110; 84.62%) and surface risk (*n* = 20; 15.38%). The remaining 36 females employed on the mine did not work directly in mining-related activities and thus were not included in our study.

In a finite population of 553 with a precision of 5% where the actual proportion of MSDs was expected to be closer to 50% with a 95% confidence interval (CI), a minimum sample size of 219 was required to give our study sufficient power, which would allow for any significant associations between the factors under study and MSD to be identified. To achieve the minimum expected sample size, we invited all 553 female mineworkers to participate in our study.

### Data collection tool and process

A self-administered questionnaire based on the Standardized Nordic Questionnaire (Kuorinka et al. 1987) and the QPSNordic General Questionnaire for Psychological and Social Factors at Work (Lindstrom et al. 2000) was used to collect data. The questionnaire covers demographic details, occupational activities, health status, exposure to physical and psychosocial factors and presence of pain symptoms. Socio-demographic details were collected on age, marital status, educational level, smoking and alcohol consumption histories and the presence of any other chronic illnesses. Female mineworkers rated their health status on a five-point scale ranging from poor to excellent and the intensity of the pain they felt from none to excruciating.

Details on occupational activities including the current occupation, whether this was underground or surface work, the number of years employed and the nature of the employment contract were collected. There were 13 questions on physical factors, 14 questions on work demands, five questions on work control, 11 questions on social interaction and one question on organisational culture. Questions on exposure to physical factors covered exposure to being involved in various static activities for an extended period (e.g. standing, sitting and squatting/kneeling in the same posture), working in awkward postures, lifting weights, exposure to vibration and involvement in repetitive activities.

Questions on psychosocial factors included questions relating to work demands, the ability to control their working activities, social interaction in the work environment and an assessment of the existing organisational culture at work. Responses to questions on physical factors, work demands, work control and social interaction were on a scale of 1 to 4 (seldom/never = 1; always = 4). Responses to the organisational culture at work were rated on a scale of 1 to 5 (rigid and rule-based = 1; encouraging and supportive = 5).

The questionnaires were translated into the common local languages spoken on the mine (isiZulu and Sotho) and back-translated to ensure that the validity of the questions was maintained. The internal consistency and content validity of the questionnaire were assessed by administering the tool to 10 female miners at the mine in September 2016 for piloting before the actual survey and the necessary corrections were implemented.

Meetings were held with the mine management and worker unions to inform them of the study. A 5-day-long workshop was held with representatives of the worker unions on the mine (National Union of Mine Workers, Association of Mine Workers and Construction Union, United Association of South Africa Solidarity). Clarity was given to the unions and workers on the study purpose and the questionnaire.

Four information-sharing sessions were held with the female mineworkers, in which they were informed about our study. During these sessions, all were invited to participate after which, signed informed consent was obtained before the issuing of questionnaires. Questionnaires were issued to participants in the language of their choice and were anonymised to reduce participation bias. The participants chose where to complete the questionnaires at their convenience (at home/work). They were given the first author’s contact details should they want clarity on any of the questions. On average, the questionnaire took an hour to complete. They returned the questionnaire to the first author who verified the completeness of their questionnaires.

### Data analysis

The data from the questionnaires were double entered into a database created in Epi-Data. The two datasets were compared to correct for any anomalies with data entry. The first author cleaned the data. Editing of the data involved checking each variable for outliers and coding errors. The data obtained from 225 employees were entered in Epi-Data and then exported to STATA V 15 for statistical analyses.

Frequencies and medians with ranges were calculated and presented for categorical and continuous demographic variables, respectively. Continuous demographic variables were categorised around the median for inclusion in bivariate and multivariable analysis. Smoking and alcohol histories were classified as having ever smoked or consumed alcohol versus having never smoked or consumed alcohol.

Female mineworkers reported several different occupation descriptions, which, with the assistance of mine management, were categorised into occupations that were ‘involved directly in mining activities’ or being ‘involved in supportive mining activities’ on the mine.

Participants’ responses to questions on physical and psychosocial factors were grouped into different factors.

Their responses to questions relating to working in static positions were summed to give the participant a total score. The total score was categorised around the median to establish low and high exposure to static positions. A similar procedure was followed for responses related to questions on repetitive movement, physical load, working in awkward postures and vibration exposure. Responses to psychosocial factors on work demands, control, social interaction and organisational culture were handled in the same manner.

The presence of pain in the neck and back in the past 12 months was based on the categorical responses of women (yes/no). Pain in the ‘upper limbs’ was determined by women’s categorical responses to pain in either shoulder, elbow or wrist and pain in the ‘lower limbs’ was determined by women’s responses to pain in either hip/thigh, knee or ankle. If a participant reported having pain in any anatomical site of the body in the past 12 months, then they were classified as having ‘pain ever in the past 12 months’.

The question of pain in the past 12 months lasting for more than 3 months was poorly answered despite the piloting of the questionnaire and was not included in the analysis. Hence, an assessment of chronic pain could not be established for this study population.

In determining the ‘intensity of pain’, women who had no pain or slight pain were classified as having low pain intensity. In contrast, women with bearable pain were classified as having moderate pain intensity, and women with excruciating pain were classified as having a high intensity of pain.

Women who reported having poor or fair health status were classified as having ‘poor’ health. In contrast, responses of good health remained classified as such, and women with very good and excellent health were classified as having an excellent health status. Women reporting a chronic disease were categorised on their response (yes/no).

On descriptive analysis, frequencies and medians with ranges were calculated and presented for categorical-dependent and continuous independent exposure variables, respectively. On univariate analysis, log-binomial regression analysis was conducted (*p* ≤ 0.05). All independent variables, which were significant for univariate analysis, were included in the multivariate analysis to test for associations with the dependent variables under study, controlling for age and education. Age was categorised around the median (≥40 years), and the educational level was categorised as having ‘high school education and above’ as opposed to having no or primary education for inclusion in the multivariable model. On multivariable analysis, moderate and high pain intensity had to be combined as good and excellent health was obtained so that the model converged.

### Ethical consideration

This study received ethical approval from the Biomedical Research Committee of the University of KwaZulu-Natal (BREC REF NO: BE473/16). The mine management and worker unions gave approval for our study to take place. All women signed informed consent before their participation.

## Results

The sample composed of 250 women (45.21%) who provided signed informed consent. A total of 235 (42.49%) returned completed questionnaires. Ten (1.8%) of the questionnaires were incomplete and thus not included in our study. The results presented below are based on the analysis of these 225 questionnaires (40.69%).

### Demographic profile of women working in a gold mine in South Africa

The median age of female mineworkers participating in this study was 40 years (range: 21–61 years), with most being married or in a live-in relationship (*n* = 121, 53.78%). The majority had either completed school or obtained a post-high school education (*n* = 182; 80.89%) (Table 1). The majority were involved in direct mining activities (*n* = 138, 61.33%), worked underground in the mine (*n* = 214; 95.11%) and were in full-time employment in the mine (*n* = 221, 98.22%) with a median employment duration of 6 years (range: 1–17 years).The majority reported neither smoking (*n* = 190; 84.44%) nor alcohol consumption (*n* = 169, 75.11%). Few rated the intensity of pain which they experienced as high (*n* = 10; 10.22%). The majority perceived themselves to be in a state of excellent health (*n* = 105; 46.67%). Only 31 (13.78%) reported having a chronic illness (Table 1). Amongst these 31, the diseases reported included hypertension (*n* = 13; 41.9%), diabetes (*n* = 8; 25.8%), asthma (*n* = 2; 6.5%), mental illness (*n* = 2;6.5%), migraine (*n* = 1;3.2%), epilepsy (*n* = 1;3.2%), carpal tunnel syndrome (*n* = 1;3.2%), HIV (*n* = 1;3.2%), TB (*n* = 1;3.2%) and chronic allergic rhinitis (*n* = 1;3.2%) (not shown).

**TABLE 1:** Demographic profile and health status of female miners in a South African gold mine in 2017 (*n* = 225).

Demographic profile	Median	Range	*n*	%
**Age in years (median; range)**	40.0	21–61	-	-
**Marital status**				
Single	-	-	74	32.89
Married/living together	-	-	121	53.78
Separated/divorced/widowed	-	-	30	14.35
**Educational level**				
Did not attend school	-	-	9	4.00
Completed primary school	-	-	34	15.11
Completed high school	-	-	87	34.67
Post-high school	-	-	95	46.22
**Current job title**	-	-	-	-
Involved directly in mining	-	-	138	61.33
Involved in supportive mining activities	-	-	87	38.67
**Years of employment (median; range)**	6.0	1–17	-	-
**Smoking status (yes)**				
Ex			26	11.56
Current	-	-	9	4.00
Never	-	-	190	84.44
Years smoked (median; range)	3.5	3–9	-	-
Amount per day (median; range)	6.0	2–10	-	-
**Alcohol consumption (yes)**				
Ex	-	-	49	21.78
Current	-	-	7	3.11
Never	-	-	169	75.11
Years consumed alcohol (median; range)	4.0	1–14	-	-
Amount per day of bottles (median; range)	5.5	2–12	-	-
**Rating of pain intensity**				
Low intensity	-	-	150	66.67
Moderate intensity	-	-	52	23.11
High intensity	-	-	23	10.22
**Health status†**				
Poor	-	-	55	24.44
Good	-	-	65	28.49
Excellent	-	-	105	46.67
**Presence of chronic diseases**	-	-	31	13.78

† , Defined on a rating scale.

### Musculoskeletal pain in the past 12 months

Approximately 50.0% (*n* = 113) reported having pain in the past 12 months. The presence of pain in the past 12 months in various sites of the body ranged from 35.11% (*n*−79) in the neck to 41.72% (*n* = 94) in the upper limbs (Table 2).

**TABLE 2:** Self-reported pain in the past 12 months amongst female miners in a South African gold mine in 2017 (*n* = 225).

Pain in the past 12 months	No		Yes	
	*n*	%	*n*	%
Neck	146	64.89	79	35.11
Back	133	59.11	92	40.89
Upper limbs	131	58.22	94	41.72
Lower limbs	142	63.11	83	36.89
Pain ever in the past 12 months	112	49.78	113	50.22

### Physical and psychosocial factors

The majority reported having high exposure to all of the physical and psychosocial factors. One hundred and forty-three (63.6%) and 146 (64.9%) reported having high exposure to repetitive movement and vibration in their work, respectively. One hundred and twenty-six (56.0%) and 157 (69.8%) reported having high work control and good organisational culture in their work, respectively (Table 3).

**TABLE 3:** Exposure to physical and psychosocial factors amongst female miners in a South African gold mine in 2017 (*n* = 225).

Variables	Low exposure		High exposure	
	*n*	%	*n*	%
**Physical risk factors**				
Working in static positions	102	45.3	123	54.7
Repetitive movement	82	36.4	143	63.6
Physical load	106	47.1	119	52.9
Working in awkward postures	85	37.8	140	62.2
Vibration exposure	79	35.1	146	64.9
**Psychosocial risk factors**				
Work demands	106	47.1	119	52.9
Work control	99	44.0	126	56.0
Social interaction	101	44.9	124	55.1
Organisational culture (good)	68	30.8	157	69.8

### Crude and adjusted associations of demographic profiles, physical and psychosocial factors, health status and the presence of pain

Having a high school education or higher was protective against reports of 12-month pain in the neck (Relative Risk (RR): 0.61; 95% CI: 0.43–0.88) and upper limbs (RR: 0.65; 95% CI: 0.48–0.89). High exposure to repetitive movements during work was significantly associated with neck pain (RR: 1.81; 95% CI: 1.17–2.81) and back pain (RR: 1.46; 95% CI: 1.01–2.09) in the past 12 months. High exposure to carrying physical loads was significantly associated with neck pain (RR: 1.62; 95% CI: 1.11–2.37), back pain (RR: 1.45; 95% CI: 1.04–2.01) and pain ever in the past 12 months (RR: 1.35; 95% CI: 1.03–1.76). Having high control over one’s work was protective against upper limb pain in the past 12 months (RR: 0.69; 95% CI: 0.51–0.94) and lower limbs (RR: 0.70; 95% CI: 0.50–0.98). Having good organisational culture at work was protective against pain in the past 12 months in the neck (RR: 0.57; 95% CI: 0.41–0.81), back (RR: 0.64; 95% CI: 0.47–0.87), upper limbs (RR: 0.67; 95% CI: 0.49–0.90) and pain ‘ever’ (RR: 0.71; 95% CI: 0.55–0.91). Having a chronic illness and reports of experiencing a moderate or high intensity of pain were significantly associated with all outcomes of pain under study. Female mineworkers’ perceptions of being in a state of good to excellent health were protective against all pain outcomes (Table 4).

**TABLE 4:** Crude associations of demographics, physical and psychosocial factors, health status and pain in the past 12 months amongst female miners in a South African gold mine in 2017.

Variable	Pain in the past 12 months														
	Neck			Back			Upper limbs			Lower limbs			Ever		
	RR	95% CI	*p*	RR	95% CI	*p*	RR	95% CI	*p*	RR	95% CI	*p*	RR	95% CI	*p*
**Demographic profile**															
Age ≥ 40 years (yes)	0.91	0.64–1.30	0.606	0.81	0.59–1.11	0.184	1.00	0.73–1.36	0.993	0.91	0.64–1.28	0.578	0.83	0.64–1.07	0.157
Married/living together (yes)	0.80	0.56–1.14	0.210	0.89	0.66–1.23	0.500	1.16	0.85–1.59	0.353	1.07	0.76–1.51	0.710	0.94	0.72–1.22	0.636
Education (high school and above)	0.61	0.43–0.88	0.008	0.75	0.53–1.06	0.105	0.65	0.48–0.89	0.008	0.70	0.48–1.00	0.054	0.79	0.59–1.05	0.107
Working directly in mining (yes)	0.93	0.65–1.33	0.676	0.86	0.63–1.17	0.335	0.97	0.71–1.33	0.856	0.96	0.67–1.35	0.800	0.95	0.73–1.24	0.719
Employment duration (≥ 6 years)	0.96	0.68–1.37	0.839	1.03	0.75–1.40	0.877	1.02	0.75–1.39	0.884	0.92	0.65–1.29	0.620	0.96	0.74–1.24	0.737
Smoking ever (yes)	0.97	0.59–1.60	0.912	1.14	0.77–1.71	0.514	1.29	0.89–1.85	0.178	1.10	0.70–1.72	0.672	1.17	0.85–1.61	0.345
Alcohol consumption ever (yes)	0.96	0.63–1.45	0.832	132	0.95–1.83	0.094	1.09	0.78–1.54	0.61	1.09	0.74–1.60	0.660	1.30	0.99–1.69	0.053
**Physical factors**															
Working in static positions (high exposure)	1.28	0.89–1.96	0.182	1.23	0.89–1.70	0.205	1.12	0.82–1.53	0.480	1.14	0.80–1.61	0.468	1.12	0.86–1.46	0.391
Repetitive movement (high exposure)	1.81	1.17–2.81	0.008	1.46	1.01–2.09	0.043	1.35	0.95–1.91	0.090	1.33	0.91–1.95	0.143	1.28	0.95–1.71	0.100
Physical load (high exposure)	1.62	1.11–2.37	0.012	1.45	1.04–2.01	0.027	1.31	0.96–1.80	0.094	1.34	0.95–1.92	0.096	1.35	1.03–1.76	0.031
Awkward postures (high exposure)	0.94	0.65–1.35	0.738	1.09	0.78–1.51	0.626	1.07	0.78–1.48	0.676	1.02	0.71–1.45	0.919	1.03	0.78–1.34	0.850
Vibration (high exposure )	0.99	0.68–1.43	0.939	1.01	0.73–1.41	0.932	1.21	0.86–1.71	0.268	1.12	0.78–1.62	0.54	0.91	0.70–1.19	0.511
**Psychosocial factors**															
Work demands (high)	0.83	0.58–1.18	0.290	0.74–1.39	0.74–1.39	0.926	0.93	0.68–1.27	0.642	1.22	0.86–1.73	0.260	1.01	0.78–1.31	0.950
Work control (high)	0.73	0.51–1.04	0.079	0.60–1.12	0.60–1.12	0.215	0.69	0.51–0.94	0.019	0.70	0.50–0.98	0.038	0.80	0.62–1.04	0.090
Social interaction (high)	0.97	0.68–1.39	0.880	0.88–1.67	0.88–1.67	0.247	1.10	0.80–1.50	0.553	1.17	0.83–1.67	0.369	1.19	0.91–1.55	0.211
Organisational culture (good)	0.57	0.41–0.81	0.001	0.47–0.87	0.47–0.87	0.005	0.67	0.49–0.90	0.008	0.77	0.54–1.08	0.129	0.71	0.55–0.91	0.006
**Health status**															
Moderate/high pain intensity rating (yes)	4.94	2.90–8.43	< 0.001	5.42	3.27–8.99	< 0.001	3.01	2.03–4.44	< 0.001	3.78	2.38–6.02	< 0.001	3.61	2.50–5.21	< 0.001
Good/excellent rating of health status (yes)	0.29	0.21–0.39	< 0.001	0.39	0.29–0.51	< 0.001	0.38	0.29–0.50	< 0.001	0.38	0.28–0.52	< 0.001	3.61	2.50–5.21	< 0.001
Presence of other chronic diseases (yes)	1.98	1.40–2.81	< 0.001	1.74	1.26–2.39	0.001	1.58	1.14–2.21	0.006	1.73	1.21–2.48	0.003	1.60	1.24–2.07	< 0.001

RR, relative risk; CI, confidence interval.

On multivariable analysis, having a high school or post-high school education was protective against reporting pain in all sites in the previous 12 months. Female mineworkers who rated their pain intensity as moderate or high were significantly more likely to report pain in all sites and ‘ever’ in the past 12 months. Being in good to excellent health was protective against reporting pain in any site or ‘ever’ in the previous 12 months (Table 5).

**TABLE 5:** Multivariable logistic regression models of demographics, physical and psychosocial factors, health status and pain in the past 12 months amongst female mine workers employed in a South African gold mine (*n* = 225).

Variable	Pain in the past 12 months														
	Neck			Back			Upper limbs			Lower limbs			Ever		
	RR	95% CI	*p*	RR	95% CI	*p*	RR	95% CI	*p*	RR	95% CI	*p*	RR	95% CI	*p*
**Demographic profile**															
Age ≥ 40 years (yes)	0.82	0.58–1.14	0.233	0.81	0.60–1.09	0.158	0.90	0.67–1.21	0.494	0.84	0.60–1.87	0.33	0.83	0.65–1.06	0.13
Education (high school and above)	0.51	0.36–0.73	< 0.001	0.66	0.48–0.90	0.009	0.61	0.44–0.84	0.003	0.63	0.44–0.90	0.01	0.71	0.54–0.94	0.12
Physical factors															
Repetitive movement (high exposure)	1.28	0.83–2.00	0.27	1.09	0.75–1.57	0.66	-	-	-	-	-	-	-	-	-
Physical load (high exposure)	1.17	0.83–1.68	0.37	1.05	0.77–1.42	0.74	-	-	-	-	-	-	1.06	0.84–1.33	0.62
**Psychosocial factors**															
Work control (high)	-	-	-	-	-	-	0.79	0.60–1.04	0.10	0.80	0.59–1.09	0.159	-	-	-
Organisational culture (good)	0.93	0.70–1.22	0.59	0.95	0.74–1.21	0.66	0.94	0.70–1.25	0.65	-	-	-	0.95	0.76–1.19	0.67
**Health status**															
Moderate/high pain intensity rating (yes)	3.20	1.81–5.64	< 0.001	4.25	2.48–7.29	< 0.001	2.24	1.46–3.44	< 0.001	2.91	1.74–4.85	< 0.001	2.96	2.00–4.27	< 0.001
Good/excellent rating of health status (yes)	0.42	0.29–0.62	< 0.001	0.61	0.45–0.84	0.003	0.52	0.38–0.72	< 0.001	0.57	0.39–0.81	0.002	0.67	0.52–0.86	0.002
Presence of other chronic disease (yes)	1.08	0.79–1.49	0.62	1.07	0.81–1.44	0.6	1	0.75–1.35	0.97	1.11	0.78–1.57	0.56	1.09	0.87–1.36	0.47

RR, relative risk; CI, confidence interval.

## Discussion

Our study documents the presence of musculoskeletal pain in the previous 12 months in females working in a mine in South Africa and highlights the need for surveillance of this condition in this working population. Importantly, it highlights that the physical and psychosocial factors associated with pain in female mineworkers are not different from those of their male counterparts (Widanarko et al. 2015a).The12-month frequency of pain reported was somewhat lower than that reported in studies of male miners (Bio et al. 2007; Sarikaya et al. 2007). The frequency of pain in the neck, back and lower and upper limbs was all less than 50%. Xu et al. (2012a) reported self-reported pain in the lower back of 64.9% amongst coal miners in China (Xu et al. 2012a), whilst Sarikaya et al. (2007) reported lower back pain in underground miners in Turkey to be as high as 78% over 5 years. On the African continent, Bio et al. reported lower back pain of 67.2% amongst miners in Ghana (Bio et al. 2007).

Occupational exposure to physical factors, including heavy physical loads, repetitive activities and vibration in underground mining, is well documented (Donoghue 2004). Dias (2014), in her study of male miners working in South African coal, gold and platinum mines, reported very high exposure to physical factors (Dias 2014). Similarly, in our study, participants reported high exposure to all physical factors confirming that the nature of their exposure is not different from males working in mining in South Africa. Whilst our participants reported that they experienced high job demands in their work, they also reported having high control of their work, good organisational culture and social interaction (support) in their work environment. These findings differ from the evidence in the literature where women tend to experience victimisation and harassment in the work environment and tend to have little control over their work (Pienaar, Naidoo & Malope 2018).

The participants who were better educated were less likely to report experiencing pain in the previous 12 months. It could be that they were less likely to be involved in activities that exposed them to high physical load and repetitive activities such as being team supervisors despite being underground. The relationship between education and occupational exposure to physical factors has been described previously. Leclerc et al. found education to be a predictor of physical work exposure, which was associated with occupational exposure (Leclerc et al. 2009). Less-educated workers fill the lower levels of jobs with high physical risk, as would also be the case with our study population.

Significant associations between high exposure to repetitive movements and physical load and neck and back pain were reported on bivariate analysis. They remained associated but not significant on multivariable analysis. These factors have been reported as risks for neck and back pain in various working populations, including mining (Andersen et al. 2002; Bio et al. 2007; Xu et al. 2011). Most of our study population were involved in underground mining activities, resulting in high exposure to physical factors. This would explain the association with pain in the neck and back in the previous 12 months.

Having high control at work has been shown to be protective against reporting musculoskeletal pain (Xu et al. 2012b; Zamri, Moy & Hoe 2017). Importantly, in our study, female mineworkers who reported having high work control were significantly less likely to report having pain in the upper limbs in the past 12 months on bivariate analysis. Xu et al., in a study of Chinese coal miners, showed that miners with low job control were 1.6 times more likely to have reported a work-related MSD when compared to other miners (Xu et al. 2012b).

In a similar vein, on bivariate analysis, female mineworkers who felt they had a good organisational culture at work were less likely to report having pain in the past 12 months, which is in keeping with other reports. Individuals working in an environment, in which there is a good organisational culture and support, are less likely to report illness. Widanarko et al. (2014), in a telephonic survey of workers in New Zealand, found a reduction in reported musculoskeletal symptoms amongst participants in the presence of good organisational work conditions. On the other hand, Fjell et al. (2007), in their study of public sector employees in Sweden, reported a significant association between poor leadership styles and musculoskeletal pain amongst males and females (Fjell et al. 2007).

Studies have shown that female mineworkers are more likely to report having a high intensity of pain as compared to males (De Cassia Pereira Fernandes et al. 2016; Rollman & Lautenbacher 2001). However, whether there is a difference in the report of pain intensity between gender groupings needs exploration. In our study, participants who perceived their pain to be of a moderate to high intensity were significantly more likely to report pain ‘ever’ and pain in all anatomical sites in the previous 12 months, compared to those who rated their pain intensity as low.

People who perceive themselves to be in poor health are more likely to report illness (Goldberg et al. 2001; Wu et al. 2013). This association is supported by our findings in which those who felt they were in good health were significantly less likely to report having experienced pain ‘ever’ or in any anatomical site in the past 12 months. A possible reason for this finding is that those in poor health may relate their health status to the pain, which they experience and, thus over-report the presence of pain. In contrast, those in good health are unlikely to focus on pain or even to recall the presence of pain in the previous 12 months.

Globally, there is a high prevalence of comorbidities (Hajat & Stein 2018) and the South African context is no different (Lalkhen & Mash 2015). In our study, there was a significant association between having another chronic illness and the presence of pain in all anatomical sites, and ‘ever’ in the past 12 months, on bivariate analysis. This association remained on multivariable analysis although this was not statistically significant.

Associations between chronic illness and musculoskeletal pain have been reported in working populations. Dias in a study of South African male miners reported a significant association between those having a mental disorder and the presence of musculoskeletal pain (Dias 2014). Furthermore, Abdulmonem et al. in a study of female teachers in Saudi Arabia reported an increased presence of MSDs in the presence of other chronic illnesses (Abdulmonem et al. 2014).

Our findings provide valuable insights into the factors associated with pain in female mineworkers and highlight the need for continuous medical surveillance of musculoskeletal pain and associated factors in this group of working females. Unmonitored female mineworkers will present with pain and musculoskeletal-related disorders, putting pressure on an already overburdened health system for care. Preventive programmes focussed on reducing exposure to physical factors and ensuring a supportive work environment and good organisational culture, will aid in reducing the burden of pain they experience. Healthcare professionals working in an occupational setting, such as occupational health physicians and physiotherapists, can assist in developing and implementing preventive and promotive programmes. This will improve the physical health of female mineworkers and reduce the ensuing burden on the health system that will occur if they continue to present with occupationally related pain.

### Limitations of our study

Whilst the findings of our study are important, the limitations need to be considered. Our study was potentially affected by the reduced participation rate, with approximately 41% of the target population participating. A larger study population may have resulted in stronger significant associations on multivariable analysis. The self-report and voluntary participation could have also led to an element of information and participation bias influencing the findings. To address this in the future, one would have to request access to medical records to match exposure, medical histories and reports of symptoms.

In confining our study to current employees, the findings could have been influenced by the healthy worker effect. Workers who had left the mine because of illness would have been missed, thus biasing the findings towards the null. In choosing to use a cross-sectional study design, we limited our ability to establish causality, because exposure and disease outcomes were measured concurrently. If we had used an alternative study design, such as a cohort study, this would have assisted in establishing exposure and disease outcomes.

## Conclusion

Our study of female mineworkers working in a gold mine in South Africa found a high presence of musculoskeletal pain in the previous 12 months. Repetitive movement and physical loading were associated with the presence of musculoskeletal pain. At the same time, good organisational culture and high work control were protective against the presence of musculoskeletal pain. Despite our study limitations, these are significant findings that should be considered in context and used to inform further research in this study population. It highlights the need for the development of ongoing work-based medical surveillance programmes for female mineworkers working in mining in South Africa. Our findings should raise awareness amongst occupational health personnel and physiotherapists. We identified factors associated with the presence of pain in female mineworkers and these may be used to manage and develop interventions to address and improve the physical health of female mineworkers in South Africa.

## References

[CIT0001] Abdulmonem, A., Hanan, A., Elaf, A., Haneen, T. & Jenan, A, 2014, ‘The prevalence of musculoskeletal pain & its associated factors among female Saudi school teachers’, *Pakistan Journal of Medical Sciences* 30(6), 1191–1196. 25674106 10.12669/pjms.306.5778 PMC4320698

[CIT0002] Andersen, J.H., Kaergaard, A., Frost, P., Thomsen, J.F., Bonde, J.P., Fallentin, N. et al., 2002, ‘Physical, psychosocial, and individual risk factors for neck/shoulder pain with pressure tenderness in the muscles among workers performing monotonous, repetitive work’, *Spine (Phila Pa 1976)* 27(6), 660–667. 10.1097/00007632-200203150-00017 11884915

[CIT0003] Bandyopadhyay, A., Dev, S. & Gangopadhyay, S, 2012, ‘A study on the prevalence of musculoskeletal disorders among the coalminers of Eastern Coalfields of India’, *International Journal of Occupational Safety and Health* 2(2), 34–37. 10.3126/ijosh.v2i2.6596

[CIT0004] Benya, A, 2009, ‘Women in mining: A challenge to occupational culture in mines’, Master of Arts (Industrial Sociology), University of Witswatersrand, Johannesburg.

[CIT0005] Bio, F., Sadhra, S., Jackson, C. & Burge, P, 2007, ‘Low back pain in underground gold miners in ghana’, *Ghana Medical Journal* 41(1), 21–25. 17622335 PMC1890537

[CIT0006] Botha, D. & Cronje, F, 2015, ‘Occupational health and safety considerations for women employed in core mining positions’, *South African Journal of Human Resource Management* 13(1), 1–12. 10.4102/sajhrm.v13i1.652

[CIT0007] De Cassia Pereira Fernandes, R., Pataro, S.M.S., De Carvalho, R.B. & Burdorf, A, 2016, ‘The concurrence of musculoskeletal pain and associated work-related factors: A cross sectional study’, *BMC Public Health* 16, 628 10.1186/s12889-016-3306-4 27449935 PMC4957833

[CIT0008] De Klerk, I, 2012, ‘The pereptions of the work environment of women in core mining activities’, Master in Business Administration, North-West University, Potchefstroom.

[CIT0009] Dias, B, 2014, ‘Musculoskeletal disorders in the South African mining industry’, Doctor of Philosophy, University of the Witwatersrand, Johannesburg.

[CIT0010] Donoghue, A.M, 2004, ‘Occupational health hazards in mining: An overview’, *Occupational Medicine* 54(5), 283–289. 10.1093/occmed/kqh072 15289583

[CIT0011] Fjell, Y., Osterberg, M., Alexanderson, K., Karlqvist, L. & Bildt, C, 2007, ‘Appraised leadership styles, psychosocial work factors, and musculoskeletal pain among public employees’, *International Archives of Occupational and Environmental Health* 81(1), 19–30. 10.1007/s00420-007-0189-9 17415585

[CIT0012] Goldberg, P., Gueguen, A., Schmaus, A., Nakache, J.P. & Goldberg, M, 2001, ‘Longitudinal study of associations between perceived health status and self reported diseases in the French Gazel cohort’, *Journal of Epidemiology and Community Health* 55(4), 233–238. 10.1136/jech.55.4.233 11238577 PMC1731872

[CIT0013] Hajat, C. & Stein, E, 2018, ‘The global burden of multiple chronic conditions: A narrative review’, *Preventive Medical Reports* 12, 284–293. 10.1016/j.pmedr.2018.10.008 PMC6214883 30406006

[CIT0014] Kunda, R, 2008, ‘Prevalence of and risk factors for work-related musculoskeletal injuries (WMSIs) amongst underground mine workers in Kitwe, Zambia’, Masters of Science, University of the Western Cape, Cape Town. 10.1539/joh.11-0175-fs 23585497

[CIT0015] Kuorinka, I., Jonsson, B., Kilbom, A., Vinterberg, H., Biering-Sorensen, F., Andersson, G. et al., 1987, ‘Standardised nordic questionnaires for the analysis of musculoskeletal symptoms’, *Applied Ergonomics* 18(3), 233–237. 10.1016/0003-6870(87)90010-X 15676628

[CIT0016] Lalkhen, H. & Mash, R, 2015, ‘Multimorbidity in non-communicable diseases in South African primary healthcare’, *South African Medical Journal* 105(2), 134–138. 10.7196/SAMJ.8696 26242533

[CIT0017] Leclerc, A., Gourmelen, J., Chastang, J.F., Plouvier, S., Niedhammer, I. & Lanoe, J.L, 2009, ‘Level of education and back pain in France: The role of demographic, lifestyle and physical work factors’, *International Archives of Occupational and Environmental Health* 82(5), 643–652. 10.1007/s00420-008-0375-4 18956210 PMC2793406

[CIT0018] Lindstrom, K., Orhede, E., Elo, A., Skogstad, A., Dallner, M., Gamberale, F. et al., 2000, *User’s guide for the QPSNordic: General nordic questionnaire for psychological and social factors at work*, Nordic Council of Ministers, cop. 2000, Kobenhavn.

[CIT0019] Minerals Council of South Africa, 2018, *Women in mining in South Africa*, Pretoria.

[CIT0020] Mokotong, R, 2016, ‘The coping mechanisms of women in the mining industry’, Masters in Social Work, University of Pretoria, Pretoria.

[CIT0021] Pienaar, H., Naidoo, P. & Malope, N, 2018, ‘Labour law and the trial and tribulations of women blue-collar workers’, in A. Bosch (ed.), *South African Board for People Practices women’s report 2018*, pp. 29–35, South African Board for People Practices, Rosebank.

[CIT0022] Rollman, G.B. & Lautenbacher, S, 2001, ‘Sexual differences in musculoskeletal pain’, *The Clinical Journal of Pain* 17(1), 20–24. 10.1097/00002508-200103000-00004 11289085

[CIT0023] Sarikaya, S., Ozdolap, S., Gumustas, S. & Koc, U, 2007, ‘Low back pain’, *American Journal of Industrial Medicine* 50, 92–96. 10.1002/ajim.20417 17238134

[CIT0024] Widanarko, B, 2013, ‘Interaction between physical and psychosocial work risk factors for low back symptoms’, Doctor of Philosophy in Ergonomics, Massey University, Auckland, Wellington, NZ. 10.1016/j.apergo.2014.07.016 25151314

[CIT0025] Widanarko B., Legg S., Devereux J. & Stevenson, M, 2014, ‘The combined effect of physical, psychosocial/organisational and/or environmental risk factors on the presence of work-related musculoskeletal symptoms and its consequences’, *Applied Ergonomics* 45(6), 1610–1621. 10.1016/j.apergo.2014.05.018 24934982

[CIT0026] Widanarko, B., Legg, S., Devereux, J. & Stevenson, M, 2015a, ‘Interaction between physical and psychosocial risk factors on the presence of neck/shoulder symptoms and its consequences’, *Ergonomics* 58(9), 1507–1518. 10.1080/00140139.2015.1019936 25815974

[CIT0027] Widanarko, B., Legg, S., Devereux, J. & Stevenson, M, 2015b, ‘Interaction between physical and psychosocial work risk factors for low back symptoms and its consqeunces amongst Indonesian coal mining workers’, *Applied Ergonomics* 46, 158–167. 10.1016/j.apergo.2014.07.016 25151314

[CIT0028] Widanarko, B., Legg, S., Stevenson, M., Devereux, J. & Jones, G, 2013, ‘Prevalence of low back symptoms and its consequences in relation to occupational group’, *American Journal of Industrial Medicine* 56, 576–589. 10.1002/ajim.22116 22975808

[CIT0029] Wu, S., Wang, R., Zhao, Y., Ma, X., Wu, M., Yan, X. et al., 2013, ‘The relationship between self-rated health and objective health status: A population-based study’, *BMC Public Health* 13, 320 10.1186/1471-2458-13-320 23570559 PMC3637052

[CIT0030] Xu, G., Pang, D., Liu, F., Pei, D., Wang, S. & Liping Li, L, 2012a, ‘Prevalence of low back pain and associated occupational factors among Chinese coal miners’, *BMC Public Health* 12(1), 149 10.1186/1471-2458-12-149 22375934 PMC3328269

[CIT0031] Xu, G.X., Li, L.P., Liu, F.Y. & Wang, S, 2012b, ‘Relationships between psychosocial factors and work-related musculoskeletal disorders in coal miners’, *Zhonghua Lao Dong Wei Sheng Zhi Ye Bing Za Zhi* 30(6), 436–438. 22931771

[CIT0032] Xu, G.X., Li, L.P., Liu, F.Y., Pei, D.S. & Wang, S, 2011, ‘Musculoskeletal disorders and risk factors of workers in a coal mine’, *Zhonghua Lao Dong Wei Sheng Zhi Ye Bing Za Zh* 29(3), 190–193. 21619816

[CIT0033] Zamri, E.N., Moy, F.M. & Hoe, V.C, 2017, ‘Association of psychological distress and work psychosocial factors with self-reported musculoskeletal pain among secondary school teachers in Malaysia’, *PLoS One* 12(2), e0172195 10.1371/journal.pone.0172195 28234933 PMC5325475

